# Antibodies to Nipah or Nipah-like Viruses in Bats, China

**DOI:** 10.3201/eid1412.080359

**Published:** 2008-12

**Authors:** Yan Li, Jianmin Wang, Andrew C. Hickey, Yunzhi Zhang, Yuchun Li, Yi Wu, Huajun Zhang, Junfa Yuan, Zhenggang Han, Jennifer McEachern, Christopher C. Broder, Lin-Fa Wang, Zhengli Shi

**Affiliations:** State Key Laboratory of Virology–Chinese Academy of Sciences, Wuhan, People’s Republic of China (Y. Li, J. Wang, Y. Zhang, H. Zhang, J. Yuan, Z. Han, Z. Shi); Uniformed Services University, Bethesda, Maryland, USA (A.C. Hickey, C.C. Broder); Yunnan Institute of Endemic Diseases Control and Prevention, Dali, People’s Republic of China (Y. Zhang); Shandong University at Weihai, Weihai, People’s Republic of China (Y. Li); University of Guangzhou, Guangzhou, People’s Republic of China (Y. Wu); Commonwealth Scientific and Industrial Research Organisation Livestock Industries, Geelong, Victoria, Australia (J. McCachern, L.-F. Wang)

**Keywords:** Nipah virus, antibodies, bats, China, letter

**To the Editor:** *Hendra* *virus* (HeV) and *Nipah* *virus* (NiV), the only known members of the genus *Henipavirus,* are 2 emerging paramyxoviruses that are highly pathogenic in a variety of vertebrate animals, including humans (*1*). Since the initial discovery of the viruses in Australia and Malaysia (*2*,*3*), sporadic HeV outbreaks have been reported from 1995 to 2007 in Australia (*4*), and regular NiV outbreaks have occurred in Bangladesh (*5*) and India (*6*). Numerous frugivorous bat species (genus *Pteropus*), and some insectivous bat species have been found to be reservoir hosts of henipaviruses in Australia and Asian countries (*7*–*9*).

In this study conducted during 2004–2007, bats were trapped within their natural habitat from 10 provinces in mainland People’s Republic of China. Serum, pharyngeal, and fecal swab samples were collected and stored as described previously (*10*). An ELISA was developed to detect antibodies to the NiV nucleocapsid (N) and attachment glycoprotein (G) proteins. For confirmation, ELISA-positive samples were tested by using Western blot against a recombinant NiV G fragment (aa 71–193) fused with the maltose-binding protein. Virus neutralization tests were conducted with live NiV and HeV under Biosaftey Level 4 containment in Australia. In addition, a surrogate neutralization test was developed by using recombinant *env*^–^ HIV-1, pseudotyped with NiV G and F. RNA was extracted by using the QIA amp Viral RNA Mini Kit (QIAGEN, Hilden, Germany). Reverse transcription–PCR (RT-PCR) was performed by using primers against the NiV N gene as described previously (*3*).

In total, 692 bat serum specimens were screened for antibody to NiV N or G protein (or both) by ELISA, and 33 were positive (Appendix Table). These specimens were from 9 of the 23 bat species examined in this study. Of the 33 serum samples reactive in ELISA, 25 with sufficient quantity left were further tested by Western blot, and 17 of 25 serum samples were reactive with MBP-NiV G fusion fragment, but not with the control MBP. None of the samples inhibited entry of NiV F/G-pseudotyped virus or neutralized either HeV or NiV. No NiV-specific RNA was detected by RT-PCR among 479 fecal swab samples and 67 throat swab samples tested; therefore, virus isolation was not attempted.

This study systematically investigated NiV presence among bats in China. The detection of henipavirus antibody suggests that several bat species have been exposed to NiV or a closely related virus. The prevalence of antibody was especially prominent among *Myotis* species from Yunnan Province. Antibody was detected in samples from 3 of 4 *Myotis* species captured in the same location in 2006 and 2007. A relatively high prevalence of henipavirus antibody was also found among *Rousettus leschenaultia* samples from Hainan Province in 2007. Notably, Yunnan and Hainan are both located in southern China. Although pteropid bats are not found in China, these data suggest henipaviruses could be introduced into China by other susceptible bat species that overlap their habitat with pteropid bats in neighboring countries.

Several possibilities may explain the failure to detect neutralizing antibodies. One might be the unique immune response among those nonpteropid bats, which results in a low level of neutralizing antibodies that are difficult to detect in the current assay systems. Alternatively, and perhaps more likely, >1 Nipah-like viruses could be circulating among the bat populations sampled in this study, producing antibodies that cross-react with, but do not neutralize, the prototype Malaysian NiV virus isolate. This phenomenon has been observed previously by our group for severe acute respiratory syndrome (SARS)–like viruses in horseshoe bats, whose sera cross-reacted with, but did not neutralize, the SARS virus in humans (*10*).

Obtaining serologic evidence of viruses in bat populations is typically more successful as a screening tool than either nucleic acid based assays or virus isolation; this is likely attributable to the often low-level of virus replication, the transient nature of the infection in bats, or both. The inability to amplify NiV sequences may have been attributable to the viral RNA present among these samples being below the threshold of detection in our assay or to the absence of infection in the population at the time of sampling. In addition, the primers used in the PCR may target regions of the NiV N protein that exhibit substantial sequence divergence in a Nipah-like virus.

Bat species in the genera *Rousettus, Myotis*, *Miniopterus,* and *Hipposideros* naturally reside in trees, buildings, and caves that can be in close proximity to human residential areas, which increases the potential of transmission of zoonotic pathogens from bats to humans. The increased risk for these zoonotic infections to spread from bats to humans in areas of cohabitation is best illustrated by the repeated spillover events involving NiV in Bangladesh (*5*). Given the present initial evidence of exposure among bats in mainland China shown here, there is an urgent need to continue and expand surveillance studies for henipaviruses in China and elsewhere on the Asian continent.

## Supplementary Material

Appendix Table Detection of Nipah virus antibody among bat serum samples collected from 10 provinces in China, 2004-2007*
