# Supplementary material for: Antibodies to Nipah or Nipah-like Viruses in Bats, China
Source: Emerg Infect Dis. 2008 Dec;14(12):1974–6. doi: 10.3201/eid1412.080359 (PMC2634619; doi:10.3201/eid1412.080359)
Supplement: Appendix Table — Detection of Nipah virus antibody among bat serum samples collected from 10 provinces in China, 2004-2007* [file 08-0359_appT-s1.pdf]

Appendix Table. Detection of Nipah virus antibody among bat serum samples collected from 10 provinces in China, 2004–2007*

	No. positive/no. tested by ELISA, 2007†						No. positive/no. tested by ELISA, 2004–2006‡					
Bat species	Hainan	Guangdong	Yunnan	Hunan	Hubei	Tianjin	Yunnan, 2006	Shanxi, 2006	Guizhou, 2005	Henan, 2005	Guangxi, 2004	Hubei, 2004, 2006
Cynopterus sphinx		0/2										
Rousettus leschenaulti	5/16 (5)										0/36	
Rhinolophus pusillus	0/11	0/1							0/7		0/7	0/9
Rhinolophus affinis	0/48	1/26 (1)					0/2		0/1	0/17		
Rhinolophus luctus	0/1			0/9								0/1
Rhinolophus sinicus	0/5	1/15 (0)	0/1		0/9		0/3		0/17			0/1
Rhinolophus pearsonii			0/3								0/32	
Rhinolophus rex									0/1			
Rhinolophus macrotis												0/3
Rhinolophus ferrumequinum												0/3
Hipposideros armiger	0/11	2/10 (0)	0/20	0/1	0/12					0/4		0/5
Hipposideros larvatus	0/1	0/20										
Hipposideros pomona	0/20	1/39 (0)					0/1					
Pipistrellus pipistrellus										0/1		
Scotophilus kuhlii		0/20										
Myotis daubentoni	0/10		3/22 (1)				6/57 (ND)					
Myotis ricketti			6/38 (4)		2/11 (2)	0/35						
Myotis altarium												0/2
Myotis spp.			1/19 (1)									
Nyctalus velutinus												0/1
Miniopterus spp.			5/15 (3)		0/1		0/5	0/3	0/6			0/2
Ia io									0/7			
Megaderma lyra									0/1			

*ND, not done.
†Glycopprotein ELISA (no. positive by Western blot).
‡Nucleocapside protein ELISA (no. positive by Western blot).
